# Reversible Mechanical Regulation and Splicing Ability of Alginate-Based Gel Based on Photo-Responsiveness of Molecular-Level Conformation

**DOI:** 10.3390/ma12182919

**Published:** 2019-09-09

**Authors:** Xiaozhou Ma, Linhai He, Xingjie Wan, Shunyu Xiang, Yu Fan, Xia Xiong, Lin Gan, Jin Huang

**Affiliations:** 1 School of Chemistry and Chemical Engineering, and Chongqing Key Laboratory of Soft-Matter Material Chemistry and Function Manufacturing, Southwest University, Chongqing 400715, China; 2 Chongqing Engineering Research Center of Application Technology for 3D Printing, Chongqing Institute of Green and Intelligent Technology, Chinese Academy of Sciences, Chongqing 400714, China; 3 College of Plant Protection, Southwest University, Chongqing 400715, China

**Keywords:** photo-responsive gel, alginate gel, azobenzene, mechanical adjustment, gel splicing

## Abstract

In this study, benefiting from the sensitive molecular conformation transversion in azobenzene, a new strategy for fabricating alginate gels with the abilities of splicing and photo-responsive mechanical adjustment is reported. Firstly, a 4,4’-azobis(benzoylhydrazide) (Azo-hydrazide) linker was used to crosslink alginate physically via the electrostatic interaction between hydrazide groups and carboxyl groups. It was then shaped and transferred in situ to a chemically crosslinked gel via 450 nm light irradiation. Under the irradiation, the molecular conformation change of azobenzene in the linker was able to form covalent bonds at the crosslinking points of the gels. Furthermore, the reversible conformation transformation of azobenzene was able to induce the increase and decrease of the storage modulus under irradiation with 365 nm light and 450 nm light, respectively, while also providing gel-like mechanical properties, depending upon the irradiation time and given wavelength. Meanwhile, the results also indicated that active groups could contribute to the splicing ability of the gel and construct a hollow cavity structure. It is believed that this work could provide a versatile strategy for preparing photo-responsive gels with reversibly tunable mechanical properties.

## 1. Introduction

Alginate has been widely applied to construct customized gel structures and biocompatible gel-based structures for bioresearch [1,2,3,4]. The rich carboxyl groups of alginate are capable of crosslinking via various physical interactions and chemical bonds to prepare gels. Compared to gels crosslinked by physical interactions, covalent bond-crosslinked alginate gels possess high structural stability and are more suitable for constructing gel structures [5,6,7,8,9]. However, the photo-induced construction of alginate-based gels with mechanical strength-tuning ability, which is very useful in the tissue engineering, is still challenging, requiring photo-responsiveness of both the crosslinking process and the network structure [6,10,11,12]. For example, to prepare the injectable and photo-crosslinking alginate gel, two types of functional groups need to be modified onto the alginate molecules: the aldehyde groups and the methylene groups. Aldehyde groups are used to form Schiff bases with amine-containing macromolecules, while methylene groups can react with each other under UV irradiation [13]. In addition, in order to adjust the mechanical properties of alginate gel, the interaction between cyclodextrin (CD) and azobenzene can give rise to the alginate crosslink. However, usually, these gels can only be turned between the sol state and the gel state under light irradiation [14].

Furthermore, numerous works have been conducted to design gel networks, in which it is possible to tune the mechanical strength of covalent bond-crosslinked gels (chemically crosslinked gels) [15,16]. In general, the adjustment of gels is achieved by the integration of responsive molecular structures, functional groups, or chemical bonds into a gel network, along with changing its crosslinking density [17,18,19,20,21]. For example, a variety of methods, such as the thermal responsiveness of the molecular structure of poly(N-isopropylacrylamide) (pNIPAAm) [22,23], the pH sensitive strength change of electrostatic interaction between functional groups [24,25], and the reversible formation of covalent bonds between coumarin units, have been reported to adjust the mechanical properties of gels [26]. However, controlling the gelation process and adjusting the gel mechanical strength in the above strategies is usually realized by two isolated responsive structures, which will increase the difficulty of gel structural design.

The molecular conformation of azobenzene can be reversibly altered under the stimulation of light fields. This photoisomerization process can effectively induce the intermolecular interaction between azobenzene molecules, and thus is applied to produce photo-responsive gels via the physical crosslinking or chemical crosslinking [27,28,29]. For physically crosslinked gels, the reversible conjugation between azobenzene and CD controlled by the azobenzene photoisomerization can achieve the sol-gel transversion of the gels [30,31]. For chemically crosslinked gels, azobenzene moieties are usually integrated into the mainframe network of the gel to shift the gel mechanical strength reversibly by its photoisomerization [32,33]. Additionally, it has also been proved that the conformation state of azobenzene moieties can affect the reactivity of its side functional groups, and contribute to the formation of covalent bonds, such as amide bonds or ester bonds [34,35]. This adjustment is mainly due to the affection of azobenzene conformation to the steric hindrance of its side functional groups. In particular, *Z*-azobenzene can induce a great steric hindrance to functional groups and decrease its reactivity, while *E*-azobenzene will reduce the steric hindrance of functional groups and allow them to form covalent bonds with other groups. This has been used to control the sol-gel transversion of dynamic bond-crosslinked gel, and the polymerization process of polymers [36,37]. However, the simultaneous control of both the crosslinking process and mechanical strength of the gel by azobenzene is still difficult to realize, which is crucial for the shaping of gels and their subsequent application.

**Figure 1:**
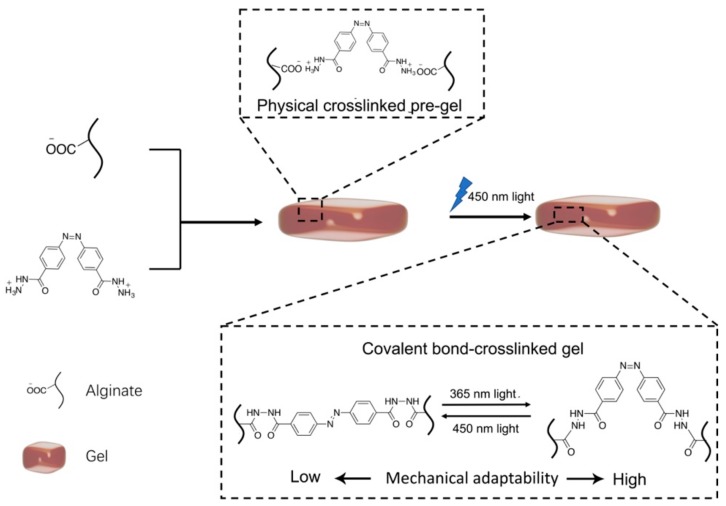
The scheme of the fabrication of Azo-alginate gel.

Herein, a new strategy for fabricating a photo-responsive chemical crosslinking and mechanically adjustable alginate-based gel (Azo-alginate gel) is reported. As shown in Figure 1, 4,4’-azobis(benzoylhydrazide) (Azo-hydrazide) functions as the linker of sodium alginate (SA). The electrostatic interaction between hydrazide groups of Azo-hydrazide and carboxyl groups of SA leads to the physical pre-crosslink of the gel. Moreover, the 450 nm light irradiation gives rise to the conformation of the Azo-hydrazide, decreases the steric hindrance of aside groups, and initiates the amidation reaction to crosslink SA via covalent bonds. Furthermore, after being crosslinked by covalent bonds, the photoisomerization of the Azo-hydrazide linker can also be used to adjust the distance of alginate molecules, altering the mechanical strength of the gel via photo irradiation. Meanwhile, unreacted hydrazide groups and carboxyl groups in the gel network can splice gel parts under the 450 nm light irradiation, leading to the construction of complex gel structures with hollow cavities. Due to a high tolerance of functional groups applied in the gel crosslinking, it is believed that this work can not only provide a versatile strategy to fabricate the crosslinking mode transformable gel, but will also show great potential of constructing a gel structure by 3D printing.

## 2. Materials and Methods

### 2.1. Materials

Sodium borohydride (99%), nitrobenzoylhydrazide (99.8%), alginage (98%) Na_2_HPO_4_ (99%), NaH_2_PO_4_ (99%) and dimethylsulfoxide (DMSO, 99.9%) were purchased form Adamas (Basel, Switzerland). N-hydroxysuccinimide (NHS, 99%) and N-ethyl-N’-(3-dimethylaminopropyl) carbodiimide hydrochloride (EDC, 99%) was purchased from J&K (Beijing, China). The double-distilled H_2_O (ddH_2_O) was purified by a Synergy (Milipore, Burlington, MA, USA). The synthesized 4,4’-azobis(benzoylhydrazide) was characterized by a JNM-ECX400S (JEOL, Tokyo, Japan). The FTIR spectra was achieved by a Nicolet iS 10 Fourier transform inferred spectrometer (ThermoFisher, Waltham, MA, USA). The mechanical properties of the gel were measured by a TA DHR rotational rheometer (TA, Newcastle, DE, USA), and 450 nm light and 365 nm light irradiation of the gel was carried by a light curing lamp equipped with 365 nm LED (20 W, 809 mW/cm^2^) and 450 nm LED (20 W, 809 mW/cm^2^) purchased from Shenzheng Bashuguang Zhaoming Co. (Shenzheng, China).

### 2.2. Synthesis of 4,4’-azobis(benzoylhydrazide)

The 4,4’-azobis(benzoylhydrazide) was synthesized according to a previous report [38]. Sodium borohydride (3.405 g, 0.09 mol) was dissolved in the 25 mL of DMSO at 85 °C. Then, 4-nitrobenzoylhydrazide (2.297 g, 0.015 mol) was dissolved in 12 mL of DMSO and added dropwise into the sodium borohydride solution for over 10 min. The mixture was reacted for 1.5 h under 85 °C, and was poured into 150 mL of water, neutralized to pH 7 with diluted HCl. The precipitate was filtered and washed by methanol and dried under 80 °C for 72 h. Afterwards, the orange precipitate was recrystallized from DMSO to obtain the pure product. The product did not melt at temperatures up to 320 °C, and the yield was 60%. The synthesized 4,4’-azobis(benzoylhydrazide) was characterized by 1D ^1^H Nuclear Magnetic Resonance spectroscopy carried out on a JNM-ECX400S (JEOL, Tokyo, Japan) operating at a ^1^H frequency of 400 MHz (solvent: DMSO-*d_6_*), 10.22–9.79 ppm (broad, 2H, CONH), 8.38–7.70 ppm (d, 8H, aromatic), 4.78–4.36ppm (s, 4H, NH_2_), 3.3 ppm (s, water) and 2.5 ppm (s, DMSO) (Figure S1).

### 2.3. Preparation of Pre-Gel

The pre-gel was prepared as follows. SA (300 mg, 4.2% w/v), EDC (271 mg) and NHS (157 mg) were added to 7 mL of phosphate buffer (PB) (20 mM, pH 6), and stirred by mechanical stirring until the SA was completely dissolved. Subsequently, 135 mg of Azo-hydrazide was dissolved in 3 mL of DMSO and added into the mixture of SA, EDC and NHS (the volume ratio of Azo-hydrazide solution and the mixture was 3:7) under vigorous stirring for 20 s. Hence, the final pre-gel (3% w/v SA) was prepared. Next, the pre-gel was poured into a dish-shaped mold with a height of 3 mm and a diameter of 2 cm to make the sample for the rheometer test. The sample was then stored in the dark to avoid light before further tests. The mechanical properties of samples were tested in air by a parallel-plate rheometer. The following modes were applied on the sample: frequency-scan (frequency range: 10%–500 Hz; strain: 5%; gap size: 3000 μm), strain-scan (frequency: 10 Hz; strain range: 0.1%–50%; gap size: 3000 μm) and time-scan (frequency: 10 Hz; strain range: 5%; duration: 1500 s; gap size: 3000 μm). The rheological test was performed at 25 °C in the dark to avoid the effect of temperature and light on the samples. All of the tests were replicated 3 times. 

### 2.4. Photo-Induced Covalent Bond Crosslinking of the Pre-Gel

The covalent bond crosslinking of the pre-gel was achieved as follows. Pre-gel (2 mL) was poured into a dish-shaped mold with a height of 3 mm and diameter of 2 cm, and was irradiated with 450 nm light for 16 h at 25 °C. The mechanical properties of the gel were then tested by a parallel-plate rheometer, and the following modes were applied to the sample: frequency-scan (frequency range: 10–500 Hz; strain: 5%; gap size: 3000 μm), strain-scan (frequency: 50 Hz; strain range: 0.1%–50%; gap size: 3000 μm) and time-scan (frequency: 50 Hz; strain range: 5%; duration: 1500 s; gap size: 3000 μm). The rheological test was performed while maintaining a temperature of below 25 °C in the dark to avoid the effect of temperature and light on the samples. All of the tests were replicated 3 times.

### 2.5. Adjustment of the Chemically Crosslinked Gel Mechanical Properties

The mechanical properties of the transformed chemically crosslinked gel were adjusted using 450 nm and 365 nm light irradiation. To study the photo-responsive mechanical change of the chemical alginate-based gel, it was firstly irradiated with 365 nm light for 0 min, 10 min, 20 min and 30 min, respectively. The storage modulus, loss modulus and tan δ of the gel were then recorded via the rheometer. Then, the gel was irradiated with 365 nm light and 450 nm light, alternately, and each irradiation lasted for 10 min or 20 min. The mechanical properties of the irradiated gel were monitored by a parallel-plate rheometer. The samples were analyzed by a time-scan rheological test with a fixed frequency and strain of 50 Hz and 5%. The rheological test was performed at 25 °C in the dark to avoid the effect of temperature and light on the samples. All of the tests were replicated 3 times.

### 2.6. Photo-Induced Gel Splicing

The gel splicing of the chemically crosslinked gel was carried out as follows. Firstly, a chemically crosslinked gel sample was cut into two pieces, and the cut surfaces of the gel parts were assembled together. The assembled gel parts were then irradiated with 450 nm light for 30 min and lifted up to test the photo-induced splicing ability of the gel. Then, to quantitatively evaluate the splicing behavior of the gel, two Azo-alginate gel samples with a height of 2 mm and a diameter of 2 cm were stacked up and irradiated with 450 nm light for 15 min. A time-scan rheological test with a fixed frequency and strain of 50 Hz and 5% was used to record the change of mechanical properties of the gel during the irradiation to evaluate the mechanical recovery degree of the gel splicing.

### 2.7. Splicing Gel Parts into Gel Structure

To prepare the chemically crosslinked gel parts for gel splicing, physically crosslinked pre-gel of Azo-hydrazide was poured into 3D-printed molds (the masses of the roof and bottom parts were 5.62 g and 3.57 g respectively), and was irradiated with 450 nm light for 30 min. The gel was then put in the dark for 16 h to completely transfer the crosslinking type of the gel, and it was then taken out of the molds to obtain Azo-alginate gel parts. The gel parts were then assembled together and irradiated with 450 nm light for 30 min to splice into the final gel structure.

## 3. Results and Discussion

### 3.1. Preparation of the Photo-Responsive Alginate Gel

**Figure 2:**
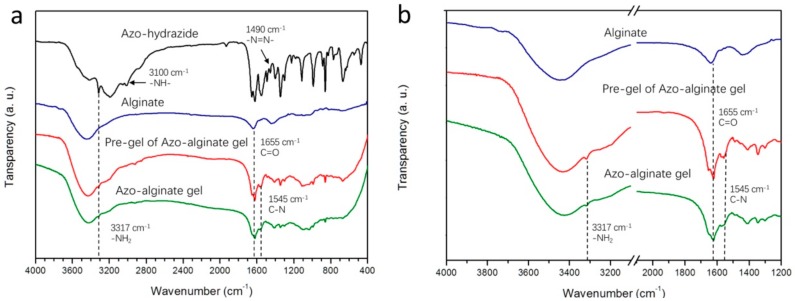
(**a**) The FTIR spectra of the Azo-hydrazide, alginate, pre-gel and Azo-alginate gel. (**b**) The FTIR spectra of the alginate pre-gel and Azo-alginate gel, showing the spectra at wavenumber ranges of 4000–3000 cm^−1^ and 2500–1200 cm^−1^.

Carboxyl groups on alginate can react with the primary amine under the catalysis of 1-Ethyl-3-(3-dimethylaminopropyl) carbodiimide (EDC) and N-Hydroxysuccinimide (NHS). This amidation reaction can be controlled by the photoisomerization of azobenzene moieties [31]. Thus, to fabricate the Azo-alginate gel, an azobenzene-based linker with primary amine groups should firstly be synthesized. Azo-hydrazide with two hydrazide groups was chosen as a photo-responsive linker for the Azo-alginate gel. The Azo-hydrazide linker was synthesized according to a previous report [38]. Its FTIR results in Figure 2a were perfectly matched with the reported FTIR spectrum of Azo-hydrazide [38]. Peaks of the Azo-hydrazide at 3317 cm^−1^ and 3100 cm^−1^ were attributed to the stretching vibration of -NH_2_ and –NH- groups, respectively. The peak located at 1490 cm^−1^ was assigned to the –N=N- of Azo-hydrazide. Meanwhile, the ^1^H NMR spectrum of Azo-hydrazide was also consistent with that of the molecule reported in the literature, indicating the successful synthesis of Azo-hydrazide [38]. 

Azobenzene moieties could be turned from an *E*- into a *Z*-conformation via irradiation with 365 nm light, and returned from *Z*- to *E*-conformation by irradiation with 450 nm light [39]. Thus, the Azo-hydrazide was firstly irradiated with 365 nm light for 30 min to control the molecular conformation of the linker, avoiding the uncontrollable amidation reaction between the linker and alginate. The pre-irradiated linker was then mixed with SA, EDC and NHS in the dark to form the pre-gel of the Azo-alginate gel. Finally, the pre-gel was irradiated with 450 nm light for 30 min and reacted for 16 h in the dark at 25 °C to form covalent bonds between the Azo-hydrazide and alginate (Figure 1). As shown in Figure 2a, the FTIR spectra of SA, pre-gel and Azo-alginate gel showed that a new amide bond was formed in the gel after the irradiation. In the pre-gel, the peak located at 1655 cm^−1^ was attributed to the C=O stretching, while the peak at 1545 cm^−1^ was assigned to the C-N stretching or N-H bending of the amide bond of Azo-hydrazide. Moreover, the peak at 1624 cm^−1^ was ascribed to the C=O groups of the alginate carboxyl groups [39] (Figure 2a). The spectrum of the Azo-alginate gel was similar to that of the pre-gel. However, it can be observed from Figure 2b that, compared with the spectrum of the pre-gel, the band at 1545 cm^−1^ was slightly strengthened, indicating the formation of a new amide bond in the Azo-alginate gel. Furthermore, the peak at 3317 cm^−1^ of the Azo-alginate gel was less intense than that of the pre-gel, suggesting the decreased number of primary amine groups in the gel. These results indicate that the irradiation with 450 nm light was able to induce the formation of amide bonds in the gel.

**Figure 3:**
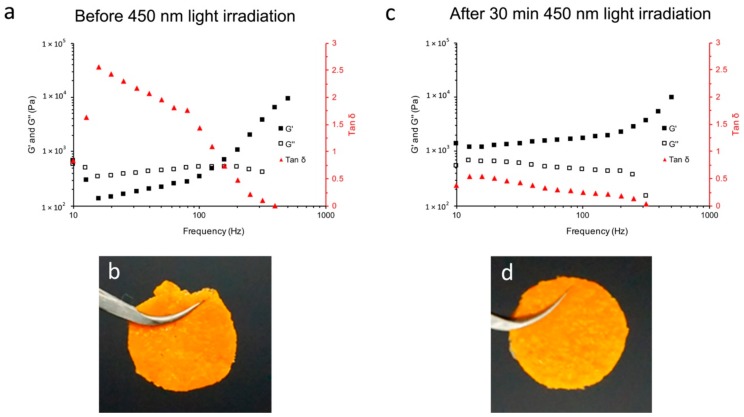
The change in rheological properties of (**a**) the pre-gel of Azo-alginate to the shearing frequency, and (**b**) its image. (**c**) The change in rheological properties of Azo-alginate gel to the shearing frequency, and (**d**) its image.

The formation of amide bonds between Azo-hydrazide and alginate would crosslink the gel chemically, and greatly enhance its structural stability, which would be reflected in the change of the rheological properties of the gel. Interestingly, after the addition of Azo-hydrazide into the alginate solution, the rheological properties of the mixture were changed. The alginate solution (3% w/v) showed a macromolecule solution-like rheological behavior. The loss modulus (G’’) of the solution was higher than its storage modulus (G’). In addition, with the increase of the shearing frequency, the solution initially presented a shearing thinning behavior, then the G’ rapidly increased to above the G’’ due to a strong interaction between alginate molecules induced by the high shearing frequency [40,41] (Figure S2a,b). However, after adding the Azo-hydrazide into the alginate solution (3% w/v) to form the pre-gel of Azo-alginate gel, the G’ and G’’ of the mixture increased, and the G’ (693 ± 52 Pa) was initially slightly higher than the G’’ (535 ± 73 Pa). Then, with the further increase of the shearing frequency, the G’ of the Azo-alginate gel decreased to lower than its G’’, indicating that the alginate solution had been transformed to a physically crosslinked gel after adding Azo-hydrazide (Figure 3a,b). At the same time, the linker pre-irradiation was also important. Owing to the affection of visible light, both *E*- and *Z*-Azo-hydrazide would exist in the linker. The pre-irradiation of the linker could turn the *E*-Azo-hydrazide in the linker into Z conformation and avoid the uncontrollable amidation reaction between alginate and Azo-hydrazide, which might make it difficult to shape the gel (Figure S2c). Furthermore, when the pre-gel was irradiated with 450 nm light to turn the Azo-hydrazide into *E*-conformation, the G’ of the gel increased obviously, while the shearing thinning behavior of the mixture could not be observed, indicating that the gel was crosslinked by covalent bonds (Figure 3c,d). This light-induced change of gel rheological properties could be utilized in the construction of the gel structure. For example, the mechanical strength and shearing thinning behavior of the pre-gel would make it flow under shearing during shaping, and provide mechanical support in the structure design. Meanwhile, the 450 nm light irradiation would improve the structural stability of the gel and thus help the structure to resist gravity.

EDC and NHS were also essential as catalysts for the light-induced mechanical property change of the gel. The mechanical property change of the mixture with or without EDC and NHS after being irradiated with 450 nm light is concluded in Table 1 and Figure S3. It could be found that the G’ and G’’ of the mixture did not change after the irradiation without EDC and NHS in the mixture of *Z*-Azo-hydrazide and SA. However, when EDC and NHS were added to the mixture, the G’ and G’’ of the pre-gel increased and slightly decreased, respectively, following the irradiation with 450 nm light, indicating the enhanced structural stability of the gel. Furthermore, when the *Z*-Azo-hydrazide/SA/EDC/NHS mixture was subjected to the 365 nm or 450 nm light field for the same duration (30 min) to control the Azo-hydrazide conformation, only the 450 nm light was able to improve the G’ of the mixture, indicating that the formation of the amide bond in the gel could be controlled by the Azo-hydrazide conformation change. On the other hand, after the pre-gel and gel were respectively immersed in ddH_2_O for 30 min, the structure of the pre-gel was completely collapsed, while the structure of the gel still remained, despite the increased volume, which proved that the covalent bond-crosslinked gel had a high structural stability (Figure S4). 

### 3.2. Affection of Linker Amount to the Gel Mechanical Strength

**Figure 4:**
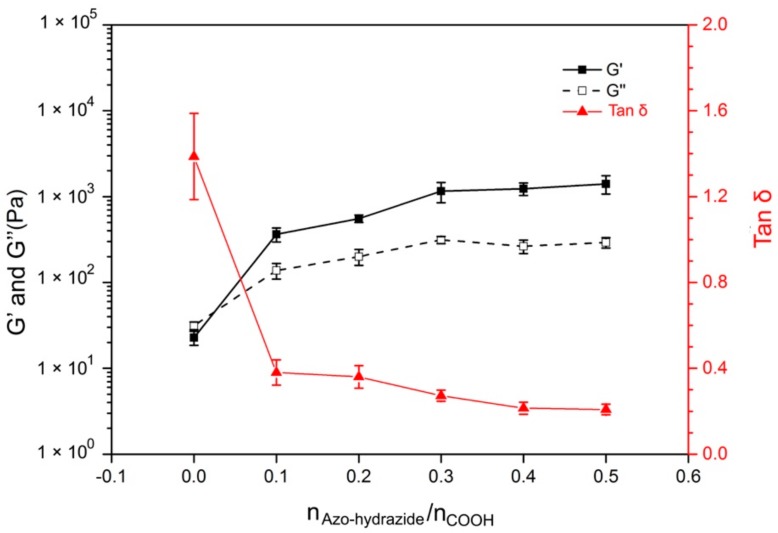
The effect of the molar ratio of linker and alginate carboxyl groups on gel mechanical properties, where n_Azo-hydrozide_ is the molar quantity of the added Azo-hydroazide, and n_COOH_ is the total carboxyl group molar quantity in the gel. All samples were analyzed by a time-scan rheological test with a fixed frequency and strain of 50 Hz and 5% respectively.

The amount of Azo-hydrazide in the Azo-alginate gel might affect the final mechanical properties of the gel, and influence the performance of the gel structure. Therefore, the effect of the molar ratio of Azo-hydrazide and total SA carboxyl groups (n_Azo-hydrazide_/n_COOH_) in the mixture on gel mechanical properties was investigated (Figure 4 and Figure S5). In Figure 4, when there was no Azo-hydrazide in the mixture, tan δ of the SA/EDC/NHS mixture was 1.38 ± 0.05, behaving as a macromolecular solution [42,43]. However, when Azo-hydrazide linkers were added to the mixture and the pre-gel was irradiated with 450 nm light, both G’ and G’’ of the pre-gel increased, revealing that the SA was crosslinked by linkers. The further increased ratio of n_Azo-hydrazide_/n_COOH_ would increase the G’ and G’’ of the Azo-alginate gel. With the increase of n_Azo-hydrazide_/n_COOH_ of the gel from 0.1 to 0.5, the G’ of the gel increased from 362.91 ± 37.44 Pa to 1408.39 ± 140.36 Pa, and the G’’ of was changed from 134.07 ± 22.64 Pa to 288.75 ± 37.22 Pa. This was primarily related to the increased number of gels. However, the tan δ of the gels decreased significantly from 0.38 to 0.21 with the increase of n_Azo-hydrazide_/n_COOH_ ratio, which was attributed to the restriction of gel network freedom by the increased number of crosslinking points. Meanwhile, it should also be noted that, compared to the reported alginate gel crosslinked by Ca^2+^, the G’ of Azo-alginate gel was higher, and was similar to that of the chemically crosslinked alginate gels [44,45].

### 3.3. Mechanical Strength Tuning of Chemically Crosslinked Gel

**Figure 5:**
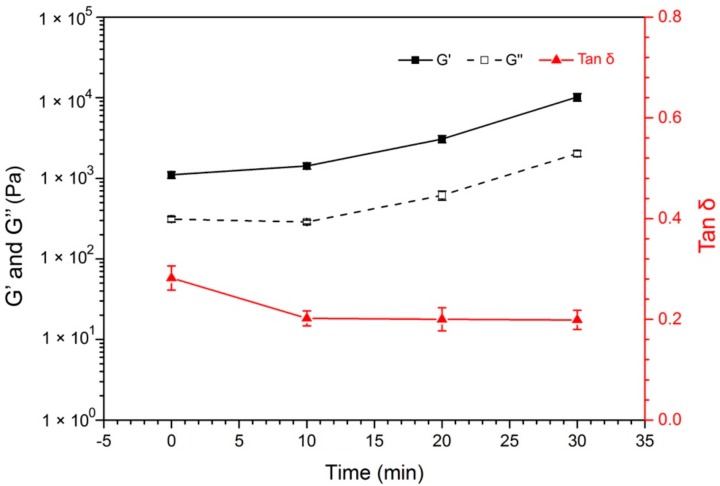
The effect of 365 nm light irradiation time on Azo-alginate gel mechanical properties.

In most photo-responsive gels, photo stimulation can only be used to control either gelation or mechanical properties of the gel [33,44,45]. However, as the photoisomerization of azobenzene moieties in the chemically crosslinked gel network could affect the gel network density [33], Azo-hydrazide could not only control the amide bond formation in the Azo-alginate gel, but also tune the gel mechanical strength under light irradiation (Figure 5 and Figure S6). As shown in Figure 5, after a freshly made Azo-alginate gel was subjected to the 365 nm light field, the gel mechanical strength increased. When increasing the exposure duration of the gel from 10 min to 30 min, its G’ and G’’ improved from 1144 ± 100 Pa to 10185 ± 1120 Pa and 316 ± 23 Pa to 2017 ± 167 Pa, respectively, due to the transformation of the *E*-Azo-hydrazide linker into the *Z* conformation. 

**Figure 6:**
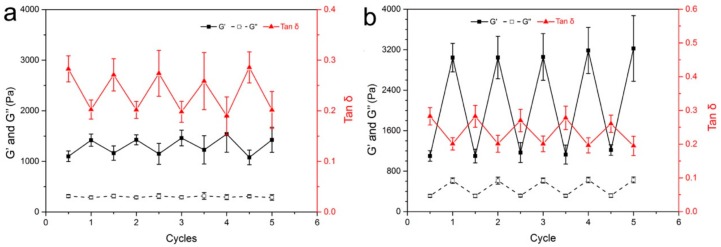
The reversible gel mechanical adjustment induced by (**a**) 10 min and (**b**) 20 min of 365 nm light and 450 nm light irradiation.

On the other hand, after the mechanical strength of the gel had been increased with 365 nm light irradiation, 450 nm light irradiation was able to relax the gel network and return its mechanical properties to their original level, owing to the transformation of the *Z*-azobenzene moieties into the *E* conformation (Figures S7 and S8). Figure 6 shows the reversible adjustment of the gel mechanical properties induced by light irradiation for 10 min and 20 min, respectively. It should be noted that even after 5 cycles of adjustment, the mechanical properties of the gel were still stable. This reversible tuning of the mechanical properties of the gel could be utilized to adjust the mechanical strength of Azo-alginate gel after shaping.

### 3.4. Photo-Induced Splicing of Chemically Crosslinked Gel

**Figure 7:**
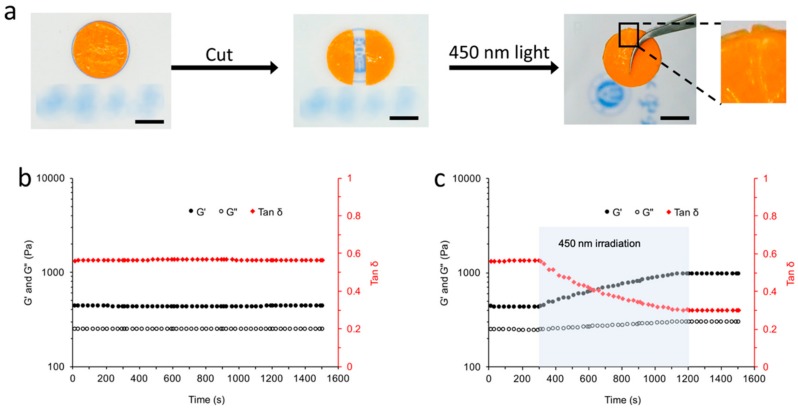
(**a**) The 450 nm light induced gel splicing. In addition, the mechanical properties of the gel stack (**b**) without and (**c**) with 450 nm irradiation.

Gel structures with hollow cavities are usually hard to construct. The production of hollow cavities in the gel is constantly hindered by the flow of the gel precursor, while the removal of excessive precursors in the cavity is also important to the quality of the resulting product. These problems could be solved by separately producing gel parts of the gel structure, followed by assembling the parts to prepare the resulting product. Due to the limitations of the gel network with respect to macromolecule movement, and the steric allowance effect given to the Azo-hydrazide linkers by the steric structure of the SA molecule, there are still some unreacted hydrazide groups in the Azo-alginate gel after the gelation. Thus, after the structural damage of the gel, these unreacted functional groups might have the ability to splice the gel parts together. To investigate the splicing ability of the Azo-alginate gel, gels with n_Azo-hydrazide_/n_COOH_ ratio of 0.3 were applied to avoid excessive hydrazide groups. In Figure 7a, a gel sample was cut into two gel parts; the cut surfaces were placed together, followed by irradiation with 450 nm light for 15 min. After that, the Azo-hydrazide gel could be spliced together and lifted up for more than 30 min. Furthermore, a method based on the literature was applied to evaluate the mechanical recoverage of the splicing [46]. Specifically, two gel samples were stacked up and irradiated with 450 nm light (Figure 7b,c and Figure S9). The G’ of the gel stack before the irradiation was lower than its original level (1019 Pa). However, when the sample was irradiated with 450 nm light, the G’ of the gel was gradually increased in 15 min, and finally maintained at 772 Pa, demonstrating the successful splicing after the light irradiation. The gel splicing would also increase its tan δ from 0.29 to 0.46, suggesting that the freedom of the gel network was higher than that of the gel before damage. This is because fewer covalent bonds were formed at the adjacent surface compared to the original level during the photo-induced gel splicing. This result indicates that complex gel structures could be built up through splicing different gel parts for their versatile splicing ability.

**Figure 8:**
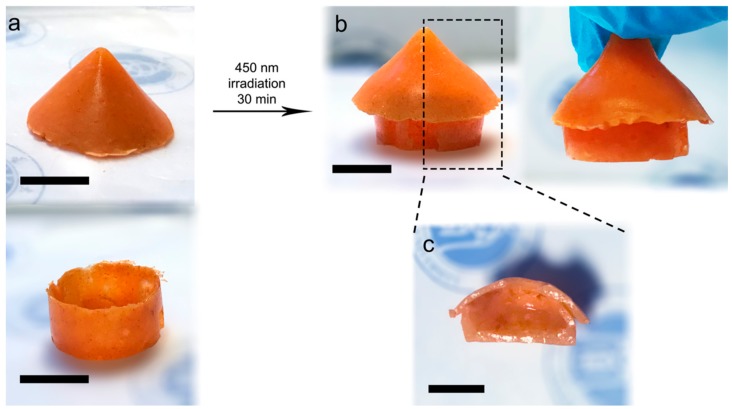
(**a**) Chemically crosslinked gel parts. (**b**) The gel structure spliced from gel parts by the 450 nm light irradiation. (**c**) Longitudinal cut surface of the gel structure, with hollow cavity. (Scale bar = 1 cm).

Based on the splicing ability of the Azo-alginate gel, a house (2.5 cm × 1.5 cm) with a hollow cavity (about 1.7 cm × 0.7 cm) was built (Figure 8). The roof (5.62 g gel) and bottom (3.57 g gel) of the house were initially built separately, and then assembled and irradiated with 450 nm light for 15 min (Figure 8a,b). After splicing, the gel house (9.19 g) could be lifted for 5 min. From the cross-sectional view of the gel house (Figure 8c), the hollow cavity of the gel structure could be supported by the gel wall. In general, gel-based structures are hard to construct due to the flow of the gel precursor solution during the gel shaping. In terms of the Azo-alginate gel, the low fluidity of the pre-gel could be of benefit to the construction of gel parts (e.g., conical roofs), avoiding the solution flow effect to the gel shape. In addition, gel splicing could also make it easier to remove excessive gel in the cavity, which favors the formation of products with high cavity qualities.

## 4. Conclusions

In this work, a new strategy for fabricating an alginate-based gel with photo-responsive splicing and mechanical adjusting ability was reported. The gel was constructed by Azo-hydrazide linkers and SA molecules. The Azo-hydrazide linkers and SA solution could quickly form a pre-gel after being mixed. Benefiting from the photoisomerization of azobenzene moieties in the linker, 450 nm and 365 nm light irradiation could be applied to prepare the covalent bond-crosslinked gel from pre-gel and reversibly adjust the gel mechanical properties. Furthermore, the remaining functional groups in the gel network could be further used to splice gel parts together under 450 nm light irradiation. The photo-induced gel splicing was then applied to construct gel structures with hollow cavities, which could avoid the gel precursor solution flowing during the synthesis process. Hence, considering the excellent tolerance of this strategy to functional groups used in gelation, it is believed that the strategy reported in this work could be used to prepare photo-responsive gels with reversibly tunable mechanical properties, which could be potentially combined with 3D printing to construct complex hollow structures with the mechanical adjustment ability. More interestingly, because the photoisomerization effect on the gel could change its mechanical properties, this Azo-alginate gel could also be applied in the adjustment of the fabrication of photo-responsive conducting materials.

## Figures and Tables

**Table 1:** The mechanical properties of the pre-gel with and without EDC, NHS and 450 nm light irradiation.

Entry	n_Azo-hydrazide_/n_COOH_	n_EDC_/n_COOH_	n_NHS_/n_COOH_	450 nm light	365 nm light	G’ (Pa)	G’’ (Pa)	Tan δ
1^2^	0.3	0	0	-	-	625.42 ± 23.53	487.46 ± 21.33	0.76 ± 0.05
2^2^	0.3	0	0	30 min	-	688.34 ± 40.29	557.38 ± 36.25	0.82 ± 0.05
3^2^	0.3	1	1	-	30 min	653.24 ± 53.33	540.20 ± 43.46	0.82 ± 0.07
4^2^	0.3	1	1	30 min	-	1542.43 ± 172.15	348.60 ± 50.44	0.23 ± 0.04

^1^ The n_Azo-hydrazide_, n_COOH_, n_EDC_, n_NHS_ are the molar quantity of Azo-hydrazide, total carboxyl groups, EDC and NHS in the pre-gel. ^2^ All the samples were analyzed by a time-scan rheological test with a fixed frequency and strain of 10 Hz and 5% respectively.

## Supplementary Materials

The following are available online at https://www.mdpi.com/1996-1944/12/18/2919/s1, Figure S1: The 1H NMR spectrum of Azo-hydrazide, Figure S2: The rheological property change of alginate solution, Figure S3: The rheological properties of the pre-gel, Figure S4: The pre-gel of Azo-alginate gel merged in ddH2O, Figure S5: The rheological properties of the gel when the molar ratio of linker and alginate carboxyl groups, Figure S6: The rheological property of chemical alginate-based gel, Figure S7: The rheological properties of Azo-alginate gel after being irradiated by 365 nm and 450 nm light for 10 min, Figure S8: The rheological properties of Azo-alginate gel after being irradiated by 365 nm and 450 nm light for 20 min, Figure S9: The G’ and G’’ of Azo-alginate gel before and after splicing.
