# Supplementary material for: Reversible Mechanical Regulation and Splicing Ability of Alginate-Based Gel Based on Photo-Responsiveness of Molecular-Level Conformation
Source: Materials (Basel). 2019 Sep 9;12(18):2919. doi: 10.3390/ma12182919 (PMC6766279; doi:10.3390/ma12182919)
Supplement: Supplementary file 1 [file materials-12-02919-s001.pdf]

materials

Supplementary

Reversible Mechanical Regulation and Splicing Ability of Alginate-Based Gel Based on Photo-Responsiveness of Molecular-Level Conformation

Xiaozhou Ma [1,2], Linhai He [1], Xingjie Wan [1], Shunyu Xiang [3], Yu Fan [1], Xia Xiong [1], Lin Gan [1] and Jin Huang [1,2,*]

[1] School of Chemistry and Chemical Engineering, and Chongqing Key Laboratory of Soft-Matter Material Chemistry and Function Manufacturing, Southwest University, Chongqing 400715, China; maxiaozhou@swu.edu.cn (X.M.), h996130557@163.com (L.H.), wxj1745193359@163.com (X.W.); fy0706@email.swu.edu.cn (Y.F.); xx362663@email.swu.edu.cn (X.X.); swucgl@swu.edu.cn (L.G.)

[2] Chongqing Engineering Research Center of Application Technology for 3D Printing, Chongqing Institute of Green and Intelligent Technology, Chinese Academy of Sciences, Chongqing 400714, China

[3] College of Plant Protection, Southwest University, Chongqing 400715, China; xiangshunyu0325@163.com (S.X.)

* Correspondence: huangjin2015@swu.edu.cn

Received: 11 July 2019; Accepted: 2 September 2019; Published: date

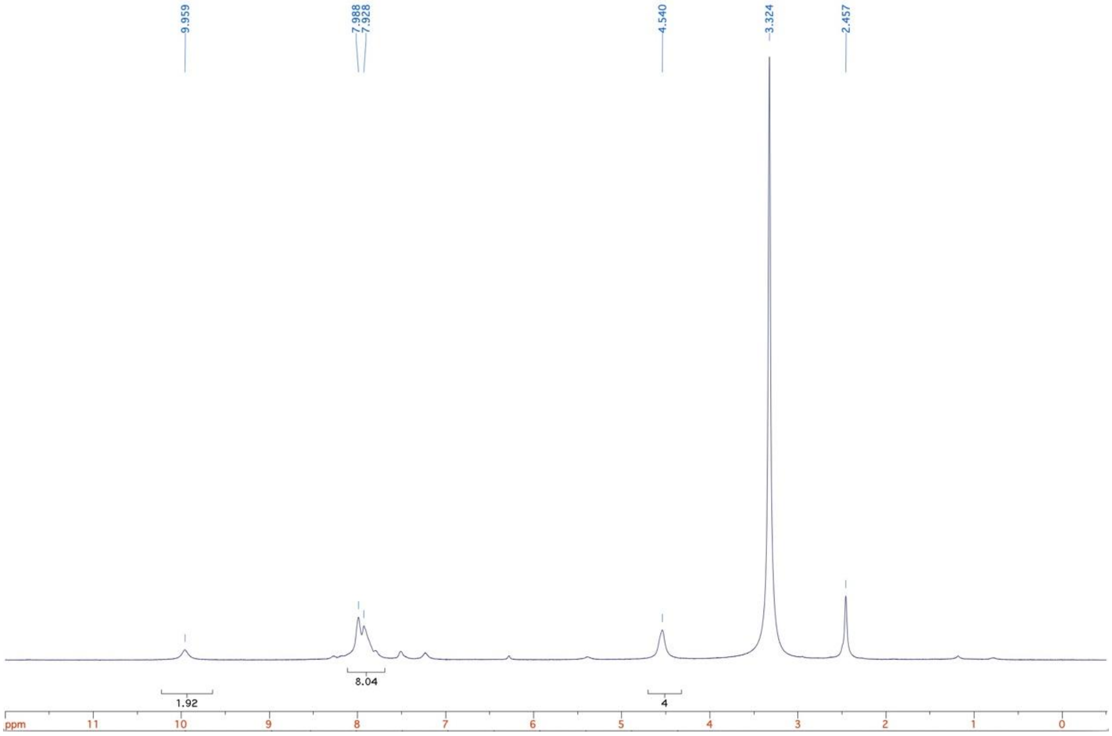

Figure S1. The ^{1}H NMR spectrum of Azo-hydrazide.

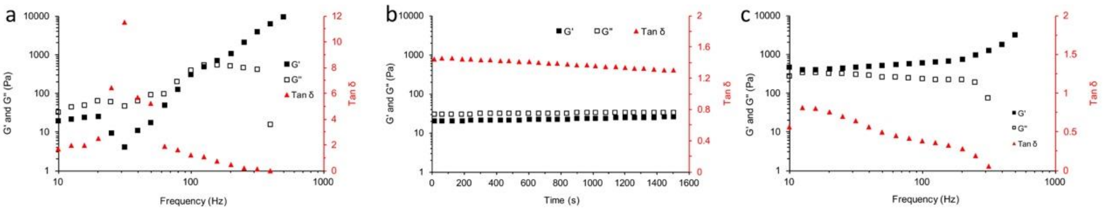

Figure S2. The rheological property change of alginate solution to (**a**) alternative shearing frequency of 10–1000 Hz (strain of 5%) and (**b**) time (frequency of 10 Hz and strain of 5%). (**c**) The rheological properties of Azo-hydrazide and alginate without pre-irradiation, the sample was analyzed by a rheological test with an alternative frequency of 10-1000 Hz and fixed strain of 5%.

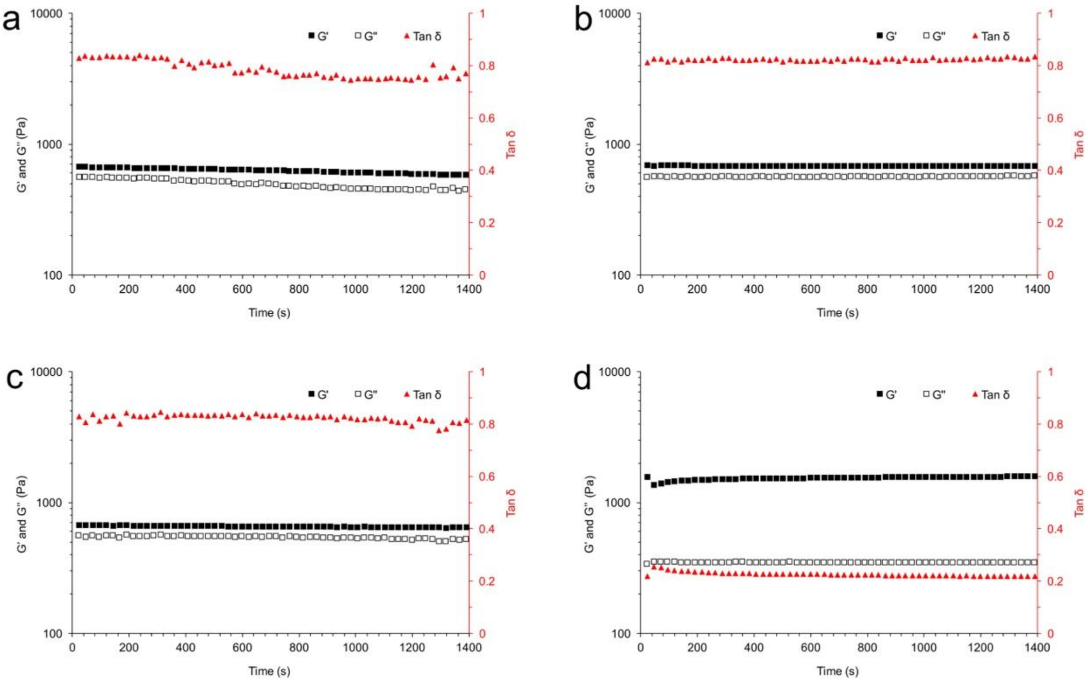

Figure S3. (**a**) The rheological properties of the pre-gel without EDCI and NHS before the irradiation of 450 nm light. (**b**) The rheological properties of the pre-gel after being irradiated by 450 nm light for 30 min. (**c**) The rheological properties of the pre-gel with EDCI and NHS after being irradiated by 365 nm light for 30 min. And (**d**) The rheological properties of the pre-gel with EDCI and NHS after being irradiated by 450 nm light for 30 min. All the samples were analyzed by a time-scan rheological test with a fixed frequency of 10 Hz and strain of 5%.

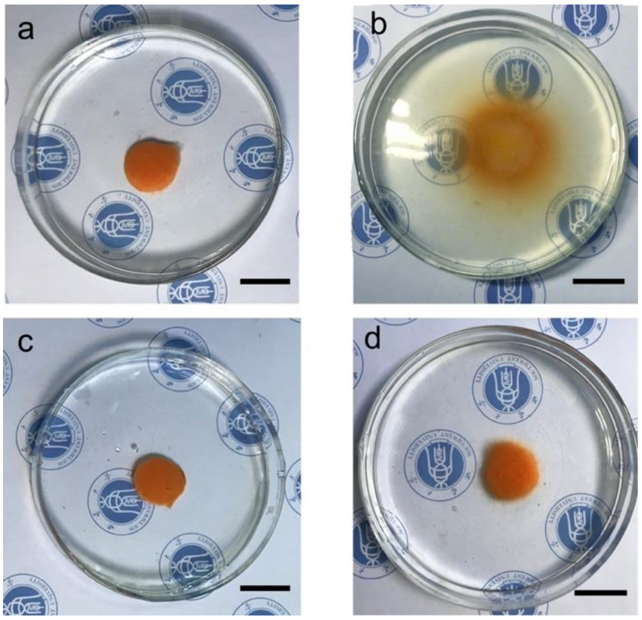

Figure S4. The pre-gel of Azo-alginate gel merged in ddH2O for (**a**) 0 min and (**b**) 30 min. The Azo-alginate gel merged in ddH2O for (**c**) 0 min and (**d**) 30 min. Scale bar = 2 cm.

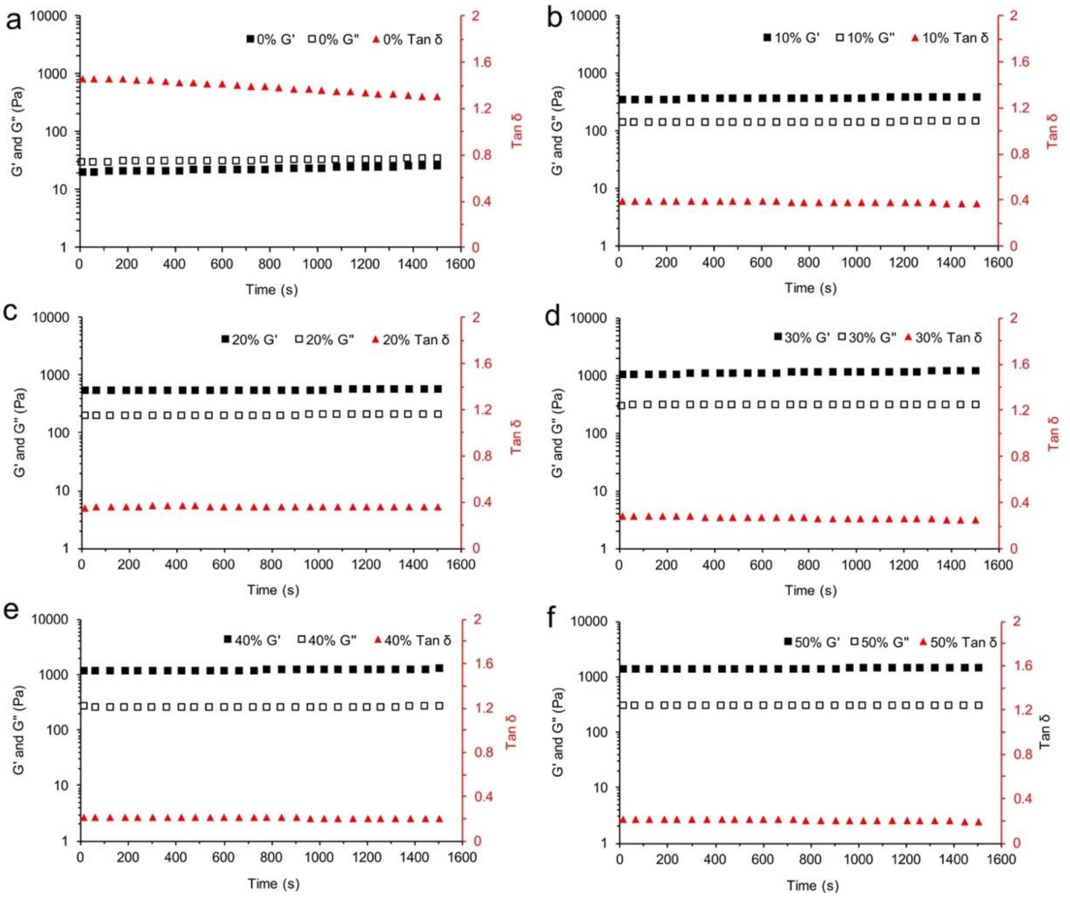

Figure S5. The rheological properties of the gel when the molar ratio of linker and alginate carboxyl groups was (**a**) 0, (**b**) 0.1, (**c**) 0.2, (**d**) 0.3, (**e**) 0.4 and (**f**) 0.5.

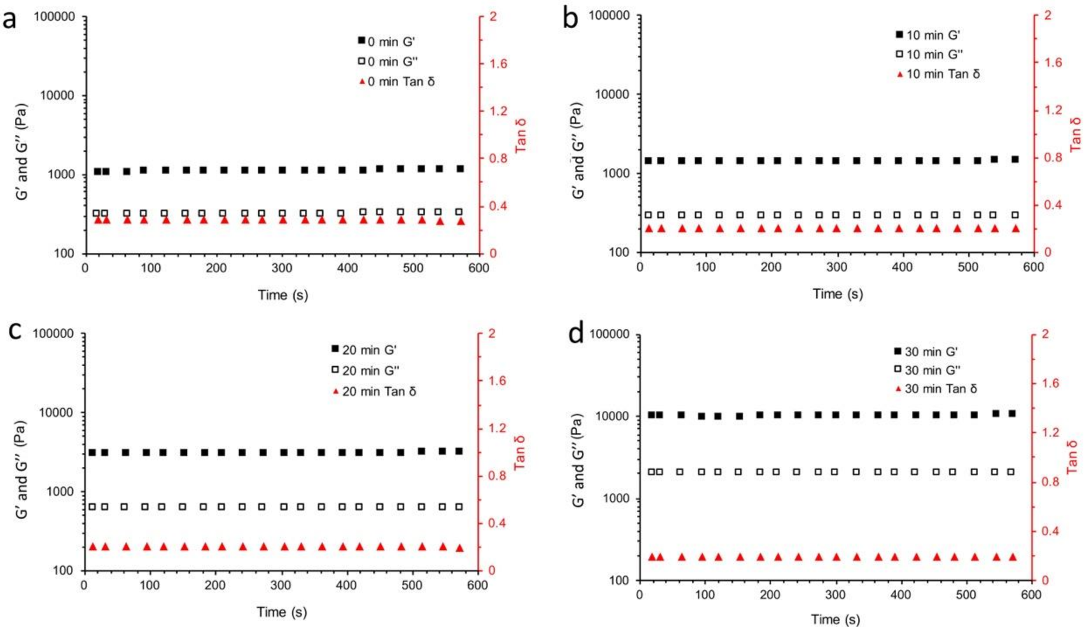

Figure S6. The rheological property of chemical alginate-based gel after (**a**) 0 min, (**b**) 10 min, (**c**) 20 min, and (**d**) 30 min 450 nm light irradiation.

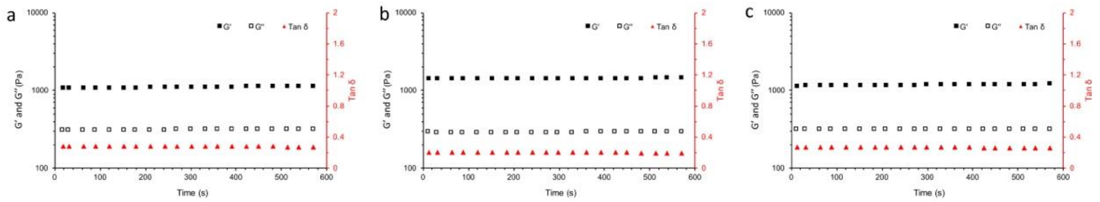

Figure S7. (**a**) The original rheological property of Azo-alginate gel. And (**b**) the rheological property of Azo-alginate gel after 365 nm light and (**c**) 450 nm light irradiation for 10 min respectively.

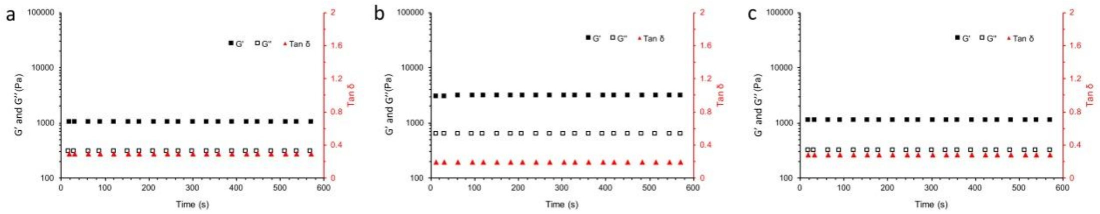

Figure S8. (**a**) The original rheological property of Azo-alginate gel. And (**b**) the rheological property of Azo-alginate gel after 365 nm light and (**c**) 450 nm light irradiation for 20 min respectively.

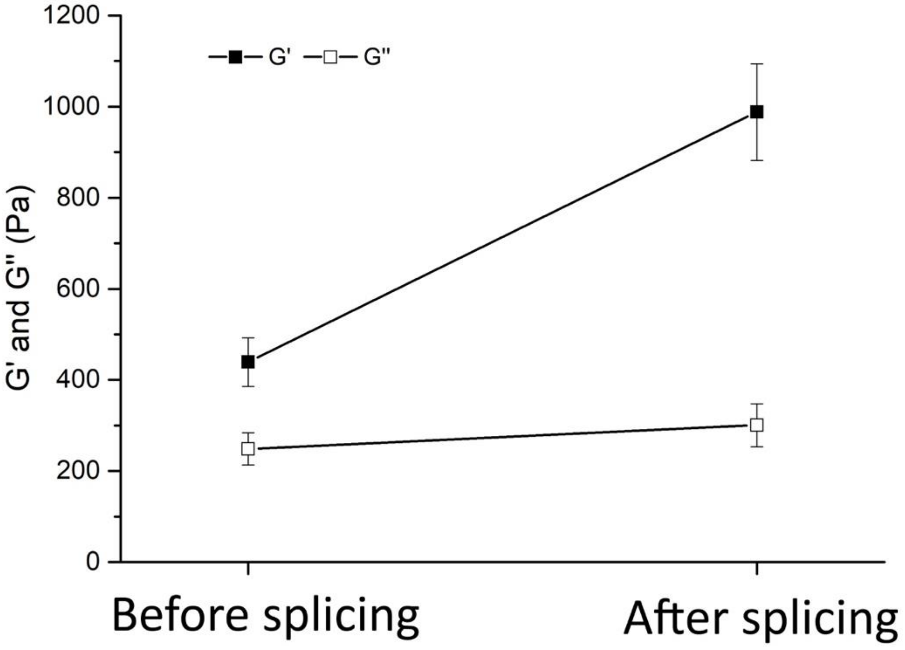

Figure S9. The G′ and G′′ of Azo-alginate gel before and after splicing. The gel was spliced by the 450 nm light irradiation for 15 min, and was analyzed by a time-scan rheological test with a fixed frequency and strain of 50 Hz and 5% respectively.

© 2019 by the authors. Submitted for possible open access publication under the terms and conditions of the Creative Commons Attribution (CC BY) license (http://creativecommons.org/licenses/by/4.0/).
